# Liver Transplantation for Hepatocellular Carcinoma among African Americans in the United States

**Published:** 2012-05-01

**Authors:** A. Sourianarayanane, F. Aucejo, C. Miller, R. Lopez, N. N. Zein, A. J. McCullough, K. V. N. Menon

**Affiliations:** 1 *Gastroenterology and Hepatology, *; 2 *Transplant Surgery, *; 3 *Quantitative Health Sciences, Cleveland Clinic, Cleveland, Ohio, USA*

**Keywords:** Liver transplantation, Survival, Hepatocellular carcinoma, HCC, outcomes, African Americans, HCV, Mortality

## Abstract

**Background:** There is increased prevalence of hepatocellular carcinoma (HCC) among African Americans (AA). Multicenter studies have shown advanced presentation, underutilization of treatment and decreased survival following liver transplantation (LT) among AA. However outcomes from single centers are not well reported.

**Objective:** To determine the outcome of AA undergoing LT for HCC at Cleveland Clinic, Cleveland, Ohio, between May 2007 and December 2009.

**Methods:** 245 consecutive patients undergoing evaluation and treatment for HCC within the mentioned time frame were studied, retrospectively.

**Results:** 80% of patients were male, 75.5% were Caucasian, 16.7% were AA and 7.8% were other ethnic groups. Compared to other ethnicities, AA subjects with HCC were more commonly female and were more likely to have hepatitis C virus (HCV) (83% *vs*. 51%, p<0.001). There were higher occurrence of HCV genotype 1 among AA compared to others among patients with this information (100% *vs*. 65%, p<0.001). In contrast to previous reports, there was no significant difference between the groups in terms of clinical presentation or management. 27% of AA underwent liver transplantation compared to 28% of the rest (p=0.88). Of the 68 patients who had LT, 9% died with no difference in post-LT survival between the two groups.

**Conclusions:** HCV (and genotype 1) is a significant risk factor for HCC in the AA population. LT results in similar survival compared to other ethnicities. AA patients with HCC benefit equally from LT compared to other ethnicities.

## INTRODUCTION

Hepatocellular carcinoma (HCC) is a highly prevalent malignancy worldwide with an estimated 748,000 new cases a year [1]. It is the second most common cause of cancer death in men and sixth in women worldwide. Recent studies suggest the age-adjusted incidence rates of HCC has increased by two folds between 1985 and 2002 in the United States [2]. The incidence of HCC is highest among Asian Americans (OR: 4.3–4.6) followed by African Americans (AA) (OR: 2.2–2.4) compared to Caucasians (OR: 1.0) [3,4]. The development of HCC in a patient with chronic liver disease is devastating and has been shown to increase mortality with a hazard ratio of 18 [5]. Viral infections are a significant risk factor for the development of HCC. While hepatitis B virus (HBV) infection is a significant risk factor for HCC among Asian Americans studies have shown hepatitis C virus (HCV) infection to be associated with HCC among Caucasians and AA in the United States [6-8].

Both the incidence of HCC and the mortality rate have been found to be higher in AA compared to the rest [9]. Studies have shown that there is under-utilization of therapies among the AA population with only 13% receiving curative therapies. Even among patients with potentially curative lesions, studies have shown that only about one-third of all patients receive adequate therapies [2]. While liver transplantation (LT) is one of the few therapies considered curative in patients with HCC complicating cirrhosis, there is under representation of AA population in waiting list for LT with only 7% in the waiting list and low numbers of patients undergoing LT for HCC [10,11]. In addition, AA patients with HCC are significantly less likely to undergo LT compared to Caucasians [11]. Following LT, survival is still poor among AA patients compared to non AA group [12]. Etiological factors and disparity in utilization of therapy combined with poor outcomes may therefore play a role in decreased survival in HCC among AA patients. While most of these studies utilize large databases comprising of patients from multiple centers, there have been few single-center studies looking at the outcome of AA patients with HCC undergoing LT.

The objectives of our study were i) to determine the outcome of AA patients undergoing LT for HCC from a single center and ii) to determine the etiology of HCC in AA.

## PATIENTS AND METHODS

Medical records of consecutive patients presenting to our tertiary care center between May 2007 and December 2009 undergoing evaluation and treatment for HCC were retrospectively reviewed. Patients with no documentation of ethnicity were excluded. We obtained demographic data including age, gender, ethnicity, etiology of underlying liver disease (cholestatic, non-alcoholic liver disease, viral hepatitis B and C, genetic and metabolic diseases), history related to smoking and alcohol, severity of underlying liver disease, tumor criteria (number and size of lesions, within or outside Milan criteria, within or outside University of California, San Francisco [UCSF] criteria), radiological and pathological evidence of vascular invasion of tumor, treatment interventions (surgery, LT, loco-regional and chemotherapy) and outcomes (recurrence and death).

All patients were seen by a dedicated team consisting of hepatologist, hepatobiliary/liver transplant surgeon, oncologist with an interest in HCC and if appropriate, by interventional radiologists. The diagnosis of HCC was made on histological or radiological criteria according to published guidelines [13,14]. Management decisions were made based on tumor criteria, the presence or absence of vascular invasion and distant metastasis and the degree of liver function. Patients with cirrhosis and HCC within Milan criteria underwent LT if found to be appropriate candidates. Loco-regional treatment was administered to these patients depending on their waiting time for LT. In patients with preserved liver function resection surgical therapy or radio-frequency ablation (RFA) was considered. The rest of the patients underwent trans-arterial chemo-embolization (TACE), bland embolization, therasphere, SIR-Sphere, cryoablation, alcohol ablation and/or sorafenib chemotherapy. A few patients underwent LT after down-staging of HCC to within Milan Criteria. The study was approved by our Institutional Review Board.

Statistical Analysis 

AAs were compared to non-AAs using *Student’s t* tests or, if appropriate, the non-parametric Wilcoxon rank sum test for continuous factors and Pearson’s χ ^2^ tests or Fisher’s exact tests for categorical variables.

Survival analysis was performed to assess pre- and post-transplant survival in each ethnic group. HCC diagnosis date was considered the first time the patient had imaging suggestive of any mass noted which resulted in diagnosis of HCC. For pre-transplant survival, time of follow-up was defined as time between HCC diagnosis and either death or censoring; subjects were censored at time of transplant or last follow-up visit if no transplant and alive.

Similarly, for subjects who received a liver transplant, post-transplant follow-up was defined as time between transplant and either death or last follow-up visit. Kaplan-Meier plots were constructed and log-rank tests were used to compare the groups. In addition, multivariable proportional hazards regression was performed to adjust for potential confounders for assessing association between ethnicity and pre-LT survival. An automated stepwise variable selection method was applied to 1000 bootstrap samples to choose the final models. Ethnic group and HCV were forced into the models and demographic and clinical characteristics that were available for 90+% of subjects were considered for inclusion; variables with inclusion rates of 50% or more were included in the model. A p<0.05 was considered statistically significant. All analyses were performed by SAS ver 9.2 software (The SAS Institute, Cary, NC).

## RESULTS

Between May 2007 and December 2009, 247 patients were evaluated with a diagnosis of HCC. Two of these patients did not have their ethnicities recorded in the medical records and were excluded from the study. The remaining 245 patients were included in the study. Two patients who died without having undergone transplantation with date of death unknown were excluded from calculation of survival analysis. The mean±SD age at time of HCC diagnosis was 64±10 years and 80% were male (Table 1). Forty-one (16.7%) subjects were AAs, of the remaining 204 patients, 185 (75.5%) were Caucasian, and 19 (7.8%) were from other ethnic groups.

**Table 1:** Demographics

	Afro-American(n=41)	Non-Afro-American(n=204)	p value
Female, n (%)	13 (31.7)	36 (17.6)	0.040
Mean±SD BMI	27.5±4.9	28.5±5.6	0.30
Smoking, n (%)			0.13
Current	13 (31.7)	42 (20.7)	
Quit	22 (53.7)	142 (70.0)	
Never	6 (14.6)	19 (9.4)	
Alcohol (current use), n (%)	7 (17.5)	16 (9.0)	0.12
Mean±SD age at time of HCC (yrs)	61.7±9.7	62.1±10.1	0.81

BMI: Body mass index (kg/m^2^); HCC: Hepatocellular carcinoma

**Figure 1:**
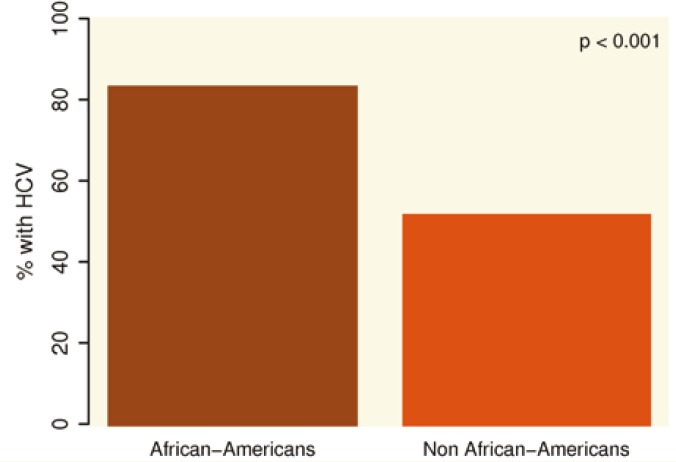
Prevalence of HCV by ethnicities among HCC patients: HCV is more common in African-Americans with HCC

Compared to other ethnicities, AA subjects with HCC were more commonly female and were more likely to have HCV (83% *vs* . 51%, p<0.001) ( Fig 1 ). HCV genotype was available for 92 of the 138 subjects with HCV. All AA patients with HCV were genotype 1 ( Table 2 ) compared to only 65% of those from other ethnic groups (p<0.001). There was no significant difference in the prevalence of HBc Ab (39% *vs* . 26%, p=0.085) or Hbs Ag positivity (5% *vs* . 5%) between the AA and non-AA groups, respectively. There were no differences in the incidence of diabetes, non-alcoholic steatohepatitis or alcoholic liver diseases in patients with HCV-related diseases between the ethnicities. There were no differences in the model for end-stage liver disease score (MELD) or Child Pugh Turcotte (CPT) scores at presentation among the two groups.

**Table 2:** Characteristics of the underlying liver disease

	Afro-American(n=41)	Non-Afro-American(n=204)	p value
Disease Etiology			
HCV	34 (82.9)	104 (51.2)	<0.001
Alcohol	6 (14.6)	56 (27.6)	0.082
NASH	1 (2.4)	27 (13.3)	0.047
HBV	2 (4.9)	11 (5.4)	0.89
Other etiologies	1 (2.4)	21 (10.3)	0.11
HCV Genotype 1	24 (100.0)	44 (64.7)	<0.001
HBsAg^+^	2 (4.9)	9 (4.6)	0.93
HBcAb^+^	16 (39.0)	51 (25.8)	0.085
Cirrhosis	38 (92.7)	183 (89.7)	0.56
Albumin	3.2±0.76	3.5±0.73	0.054
Bilirubin	1.3 [0.60, 2.4]	1.1 [0.60, 2.1]	0.49
Creatinine	0.92 [0.84, 1.2]	0.90 [0.73, 1.10]	0.088
INR	1.2 [1.00, 1.3]	1.1 [1.1, 1.3]	0.56
MELD	12.0 [8.0, 15.0]	10.0 [7.0, 13.0]	0.17
CTP scores	7.0 [5.0, 8.0]	6.0 [5.0, 8.0]	0.21

Values presented as Mean±SD; n (%); median [25%ile, 75%ile]

NASH: Non-alcoholic steatohepatitis, HCV: Hepatitis C virus; HBV: Hepatitis B virus

HBs Ag: Hepatitis B surface antigen, HBc Ab: Hepatitis B core antibody,

INR: International normalized ratio, CTP: Child Turcotte Pugh, MELD: Model for end-stage liver disease

There were no significant differences between AA and non-AA population in terms of presenting within Milan criteria (42% *vs* . 50%, p=NS), within UCSF criteria (56% *vs* . 65%, p=NS) for HCC, or radiological evidence of vascular invasion (24% *vs* . 27%, p=NS) ( Table 3 ). There was no significant difference between the number of AA patients and the rest who underwent surgical therapies (resection and/or liver transplantation) for HCC (37% *vs* . 46%, p=0.26) or liver transplantation (27% *vs* . 28%, p=0.88). There were no differences among non-surgical therapies received between the two groups (51% *vs* . 53%).

**Table 3:** Clinical presentation and treatment of HCC

	Afro-American(n=41)	Non-Afro-American(n=204)	p value
Within Milan criteria	17 (41.5)	101 (49.5)	0.35
Within UCSF criteria	23 (56.1)	132 (64.7)	0.30
Radiological vascular invasion	10 (24.4)	52 (27.4)	0.70
Pathological vascular invasion	4 (33.3)	22 (40.0)	0.67
LT	11 (26.8)	57 (27.9)	0.88
Resection	4 (9.8)	39 (19.1)	0.15
LT and/or resection	15 (36.6)	94 (46.1)	0.26
RFA and/or TACE	16 (39.0)	85 (41.7)	0.75
Other palliative therapy	11 (26.8)	36 (17.6)	0.17
Recurrence	1 (33.3)	12 (63.2)	0.33
Deceased	25 (61.0)	98 (48.0)	0.13
Pre-surgical mortality	22 (53.7)	84 (41.2)	0.14
Post-surgical mortality, if applicable	3 (20.0)	14 (14.9)	0.61
Total follow-up months	7.6 [2.6, 18.1]	10.3 [4.0, 17.5]	0.18

Values presented as Mean±SD; n (%); Median [25%ile, 75%ile]

UCSF: University of California, San Francisco; LT: Liver transplantation; LRT: Loco-regional therapy; RFA: Radio-frequency ablation, TACE: Transarterial chemo-embolization HCC: Hepatocellular carcinoma

Survival 

Overall, there was no significant difference in mortality between AA patients and the rest during follow-up (61 *vs* . 48%, p=0.13).

**Figure 2:**
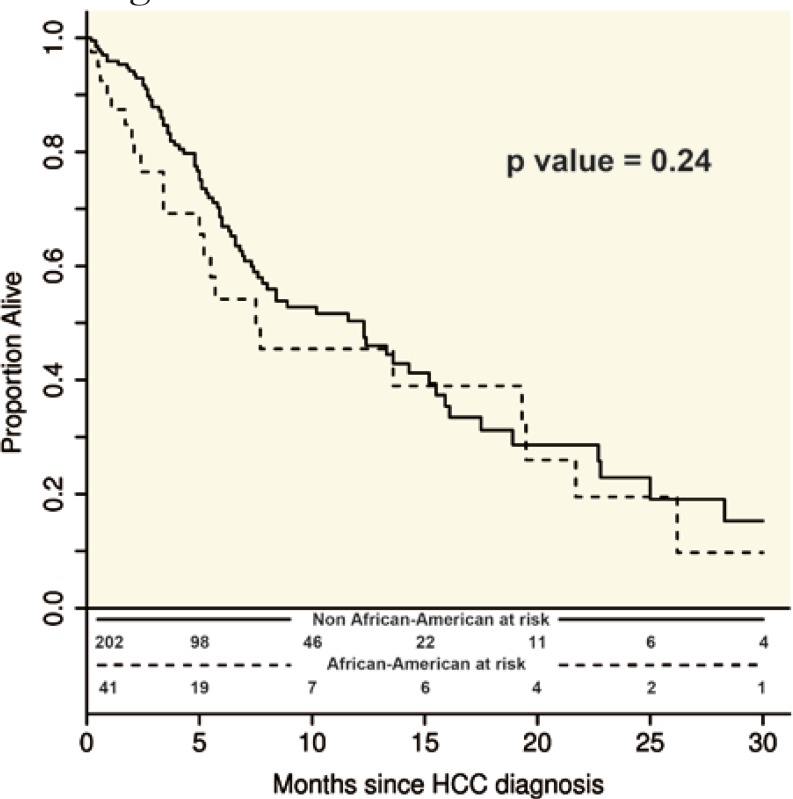
Survival of patients with HCC prior to surgical intervention

Fifty-four percent of AA patients died prior to receiving a surgical intervention (resection or LT) compared to 41% of the rest (p=0.14). The median pre-surgery survival time was 7.7 (95% CI: 5.0–19.3) months in AA compared to 12.3 (95% CI: 7.6–15.2) months in non-AA patients ( Fig 2 , p=0.24). After adjusting for HCV, vascular invasion, cirrhosis, age, MELD and UCSF criteria, there was no difference in survival (HR:1.2, p=0.52) between the two groups. In multivariate analysis ( Table 4 ), pre-surgical independent predictors of mortality were radiological evidence of vascular invasion (p<0.001), cirrhosis (p=0.012), age (p=0.005) and MELD (p<0.001).

**Table 4:** Pre-liver transplant mortality: multivariable analysis

	HR (95% CI)	p value
African-American	1.2 (0.70–2.0)	0.52
HCV	1.05 (0.64–1.7)	0.84
Radiological evidence of vascular Invasion	2.6 (1.6–4.1)	<0.001
Cirrhosis	6.2 (1.5–26.1)	0.012
Age (5-yr increase)	1.2 (1.05–1.3)	0.005
MELD (1-unit increase)	1.1 (1.1–1.2)	<0.001
Outside UCSF Criteria	1.5 (0.98–2.4)	0.061

HR: Hazard ratio; CI: Confidence interval

HCV: Hepatitis C virus; MELD: Model for end-stage liver disease; UCSF: University of California, San Francisco

**Figure 3:**
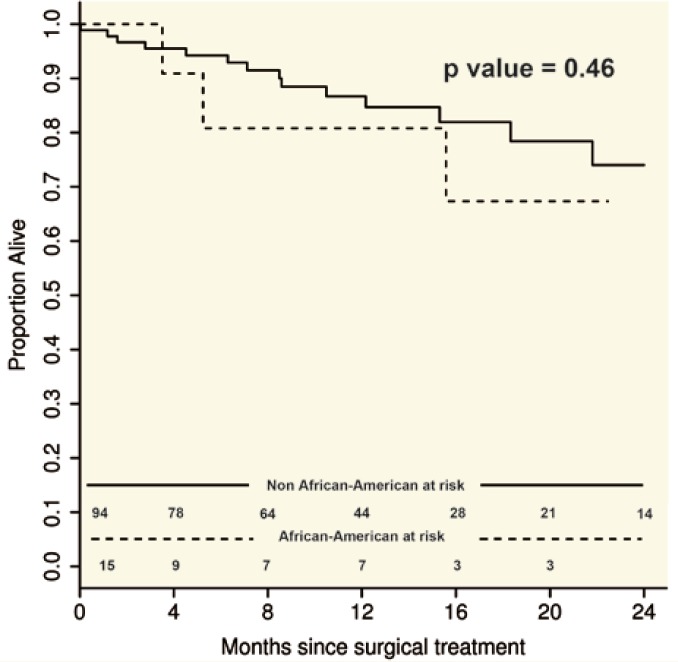
Survival of patients with HCC undergoing liver transplantation

Post-LT survival:

Twenty-seven percent (n=11) of AA had a liver transplant compared to 28% (n=57) of non-AA (p=0.88). Of the 68 patients who had LT, 9% died. There was no difference in post-LT survival between the two groups ( Fig 3 ).

## DISCUSSION

Our single center study shows no significant difference in survival rates in AA patients undergoing LT for HCC compared to the non-AA population. This is different from previous studies that have shown a decreased survival in AA patients undergoing LT for HCC [ 15 , 16 ]. Most of these studies are based on nationwide samples such as the SEERS database, UNOS database or nationwide inpatient sample (NIS) data and hence may not be truly valid with reference to individual institutions. There are only a few single institution studies looking at survival of AA patients undergoing LT for HCC. Harrison, *et al* , found no difference in the outcome of AA patients/Hispanic patients undergoing LT compared to whites [ 17 ]. In a later study by Yu, *et al,* comprising of 19 AA patients who underwent LT for HCC, there was a significant difference in overall survival by ethnicity using the log-rank test but race and insurance were not independently predictive of mortality [ 18 ]. Our study is the first one looking specifically at survival of AA patients with HCC undergoing LT and we found no difference in survival between AA patients and the rest. LT should therefore be strongly considered in AA with HCC.

Studies have shown decreased utilization of therapies such as surgery and LT for HCC among the AA, especially transplantation and surgical services [ 11 , 16 , 18 - 20 ]. These issues are thought to be partly responsible for the poor survival of AA patients with HCC. In our study, in contrast, there was no difference in utilization of various surgical therapies between AA and the rest. Additionally, late presentation with advanced disease and medical insurance differences are some of the causes attributed to the differences in utilization of resources among the AA population [ 18 , 21 ]. In our study there were no differences between AA and non-AA groups in the stage of presentation by Milan or UCSF criteria or the occurrence of radiological evidence of vascular invasion of HCC. There were also no differences in the number of treatment modalities received among ethnicities and there were no differences in mortality between these groups as a whole.

We found a higher incidence of HCV among AA compared to other ethnicities as noted in other studies [ 10 ]. This is in contrast to other studies where HCV was found to be equally prevalent in both AA patients and non-AA patients [ 7 ]. AA patients with HCV represent a specific population who may benefit from heightened screening and preventive efforts to identify and treat HCC.

In our cohort vascular invasion, the presence of cirrhosis, age and MELD score were independent risk factors for mortality. This is in keeping with previously published data where vascular invasion has been correlated with poor survival [ 22 , 23 ].

Our numbers are small when compared to larger multi-center studies. However, our single center study had a diverse population similar to national demographic data for HCC and its etiology. In our study, there were no differences between AA and non-AA population in terms of their liver and HCC disease stages and among those who underwent LT and resection. We did not find evidence to suggest late presentation with advanced disease or underutilization of resources among AA population due to medical insurance as considered by others. This allowed us to compare survival and treatment outcomes without the inherent biases of large database driven multi-center studies. Some of the improvements in survival among AA seen in our study could be due to the availability of newer treatment options and the multi-disciplinary approach offered by our institution compared to data available from national registries which includes time periods when some of the current treatment options and down-staging of HCC were not common. 

In conclusion, in our single center study, there was no difference between AA and the rest in terms of management and other clinical characteristics. HCV (genotype 1) was the predominant risk factor for HCC in the AA population. There was no difference in survival among patients who underwent LT. AA patients with HCC benefit equally from LT for HCC.

## Notes

Financial Support: **None declared.**
